# LncRNA SNHG12 contributes to multidrug resistance through activating the MAPK/Slug pathway by sponging miR-181a in non-small cell lung cancer

**DOI:** 10.18632/oncotarget.20475

**Published:** 2017-08-24

**Authors:** Pei Wang, Dong Chen, Hongbing Ma, Yong Li

**Affiliations:** ^1^ Department of Cardiothoracic Surgery, Huaihe Hospital of Henan University, Kaifeng 475000, China

**Keywords:** lncRNA, SNHG12, miR-181a, MAPK/Slug, NSCLC

## Abstract

Small nucleolar RNA host gene 12 (SNHG12), as one of the long non-coding RNAs (lncRNAs), plays an oncogenic role in various cancers, however, its role in the chemoresistance of non-small cell lung cancer (NSCLC) is unclear. In this study, we investigated the effect of SNHG12 on multidrug resistance (MDR) in NSCLC. The results showed that SNHG12 was high-expressed and miR-181a was low-expressed in NSCLC tumor tissues and cell lines. Knockdown of SNHG12 reversed the resistance to cisplatin, paclitaxel and gefitinib in A549/DDP, A549/PTX and PC9/AB2 cells through inducing cell apoptosis. Moreover, SNHG12 silencing suppressed MAPK1 and MAP2K1 expression by upregulating miR-181a, leading to inhibition of the MAPK/Slug pathway through decreasing phosphorylated MAPK1 (p-MAPK1), phosphorylated MAP2K1 (p-MAP2K1) and Slug levels. Furthermore, downregulation of SNHG12 enhanced the sensitivity of NSCLC cells to cisplatin in nude mice. Overall, our study is the first to identify a SNHG12-miR-181a-MAPK/Slug axis to elucidate in part how SNHG12 exert functions in NSCLC MDR, providing a novel therapeutic target to overcome MDR in NSCLC.

## INTRODUCTION

Lung cancer is one of the most common malignancies, and is the most frequent cause of cancer-related mortality accounting for an estimated 1.59 million deaths worldwide [1]. Approximately 80-85% of lung cancer cases are currently classified as non-small-cell lung cancer (NSCLC) [2]. At present, chemotherapy is regarded as a major supplementary therapy used to manage NSCLC following surgical operation. Chemotherapeutic agents such as cisplatin and paclitaxel, involved in platinum-based chemotherapy, and gefitinib, one of epidermal growth factor receptor (EGFR) tyrosine kinase inhibitors (EGFR-TKIs), are first-line treatments for NSCLC. However, certain patients gradually develop multidrug resistance (MDR), limiting the effectiveness of chemotherapy. Therefore, further elucidation of molecular mechanisms underlying MDR becomes indispensable in order to improve the therapeutic outcome NSCLC.

MicroRNAs (miRNAs) are small non-coding endogenous RNAs with 19 to 25 nucleotides in length that trigger either translational repression or mRNA degradation by binding to the 3’ untranslated region (3’ UTR) of their target mRNAs through incompetently base pairing [3]. Previous documents demonstrated that miRNAs are implicated in chemoresistant phenotype of diverse tumors via different mechanisms, such as anomalous regulation of apoptosis, cell cycle distribution, activity of drug efflux transporters, DNA repair and alterations in drug targets [4]. Also, considerable experimental evidence confirmed the involvement of miR-181a in drug sensitivity or resistance, functioning as either an oncogene or a tumor suppressor depending on the cancer type and/or cellular context. For instance, miR-181a attenuated the chemoresistance with inhibition of EMT and metastatic potential by targeting Twist1 in tongue squamous cell carcinoma [5]. miR-181a overexpression enhanced the sensitivity of NSCLC cells to cisplatin by stimulating Bax oligomerization and activating proapoptotic caspases [6]. In contrast, microRNA-181a induced the chemoresistance of human cervical squamous cell carcinoma via apoptosis reversion at least partly by downregulating PRKCD [7]. Upregulation of miR-181a increased the resistance of SKOV3/PTX cells to paclitaxel by promoting EMT and inhibiting paclitaxel-induced cell apoptosis in ovarian cancer [8]. However, how miR-181a is regulated and the mechanism by which miR-181a affects the drug resistance in NSCLC are largely unknown.

Long non-coding RNAs (lncRNAs) stand for a new class of non-coding transcripts consist of more than 200 nucleotides. Misregulated lncRNAs have been linked to multiple cellular processes including cell apoptosis, invasion, migration, and metastasis in various cancers [9]. In recent years, rapidly emerging evidence has confirmed the roles of lncRNAs in evaluating drug resistance or sensitivity and a large amount of researches are focusing on disclosing the precise molecular mechanism of lncRNA-regulated drug resistance [10]. Multiple studies have suggested that lncRNAs, including MALAT1 [11], CUDR [12], H19 [13], HOTAIR [14] and UCA1 [15], are involved in chemotherapy resistance of cancer cells. Small nucleolar RNA host gene 12 (SNHG12) is a lncRNA located at chromosome 1p35.3. Moreover, the MiTranscriptome database revealed that the expression level of SNHG12 was upregulated in human lung cancer [16]. However, the role and specific mechanism of lncRNA SNHG12 in NSCLC remains completely unclear. In the present study, the role and underlying molecular mechanisms of SNHG12 in chemoresistance of NSCLC were explored.

## RESULTS

### SNHG12 is upregulated and miR-181a is downregulated in NSCLC tissues and cell lines

**Figure 1:**
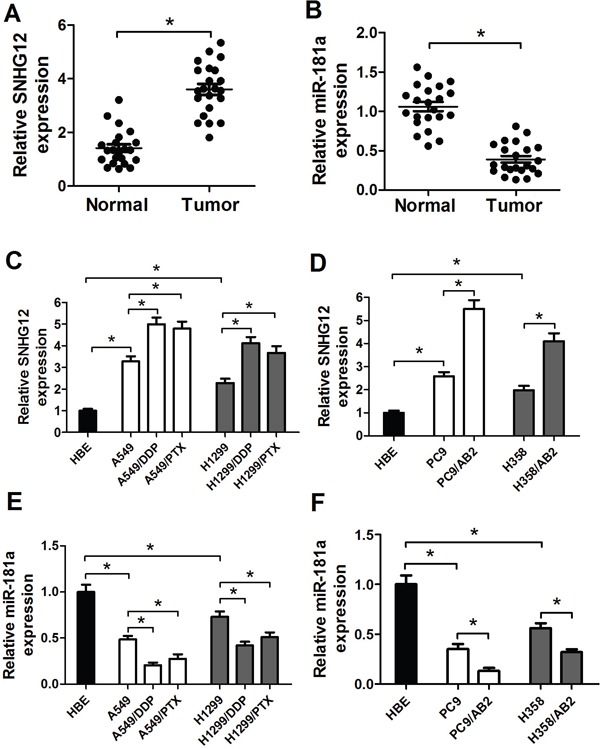
Upregulation of SNHG12 expression and downregulation of miR-181a expression in NSCLC tissues and cell lines **(A** and **B)** Expression levels of SNHG12 and miR-181a in 22 pairs of NSCLC tumor and adjacent normal tissue specimens were examined by qRT-PCR analysis. qRT-PCR analysis was performed to detect the expressions of SNHG12 **(C** and **D)** and miR-181a **(E** and **F)** in NSCLC cell lines (A549, A549/DDP, A549/PTX, H1299, H1299/PTX, H1299/DDP, PC9, PC9/AB2, H358 and H358/AB2) and immortalization of human bronchial epithelial cell line HBE. ^*^P < 0.05 versus control groups.

To ascertain the expression levels of SNHG12 and miR-181a in NSCLC tissues, qRT-PCR analysis was performed in 22 paired NSCLC tumor tissue specimens and adjacent normal tissues. Results indicated that the level of SNHG12 expression was upregulated while miR-181a expression was downregulated in tumor tissues when compared with adjacent normal tissues (Figure 1A and 1B). Then, the level of SNHG12 and miR-181a was further determined in NSCLC cell lines A549 and its drug-resistant cell strains (A549/DDP and A549/PTX), H1299 and its drug-resistant cell strains (H1299/DDP and H1299/PTX), PC9 and its gefitinib-resistant cell strain PC9/AB2, H358 and its gefitinib-resistant cell strain H358/AB2, and immortalization of human bronchial epithelial cell line HBE. qRT-PCR analysis revealed that SNHG12 expression (Figure 1C and 1D) was dramatically increased and miR-181a expression (Figure 1E and 1F) was remarkedly reduced in A549, H1299, PC9 and H358 cells when compared with HBE cells (Figure 1C-1F). Moreover, the levels of SNHG12 in A549, H1299, PC9 and H358 cells were lower than their respective resistant cell strains A549/PTX, A549/DDP, H1299/PTX, H1299/DDP, PC9/AB2 and H358/AB2 (Figure 1C and 1D). In contrast, A549/PTX, A549/DDP, H1299/PTX, H1299/DDP, PC9/AB2 and H358/AB2 cells exhibited low-expression levels of miR-181a compared with their parental cell strains A549, H1299, PC9 and H358 (Figure 1E and 1F). Considering that the higher expression of SNHG12 and lower miR-181a levels in A549/PTX, A549/DDP and PC9/AB2 cells, these cells were used for further investigations.

### SNHG12 directly suppresses miR-181a expression

**Figure 2:**
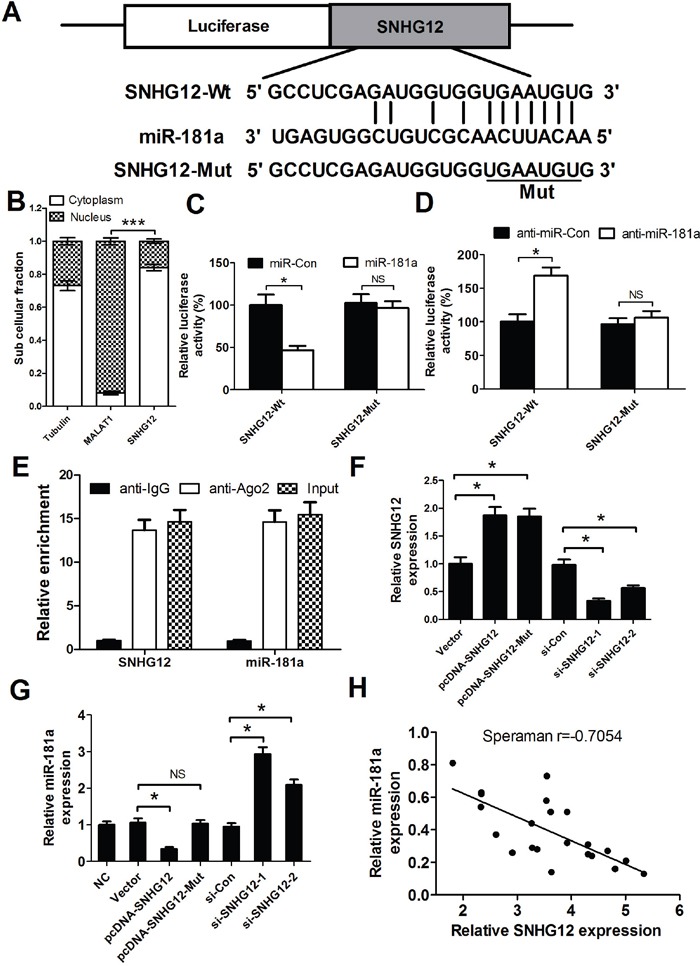
SNHG12 acts as a miR-181a sponge **(A)** The binding site of miR-181a within the SNHG12. **(B)** The levels of nuclear control transcript (MALAT1), cytoplasmic control transcript (Tubulin), and SNHG12 were determined by qRT-PCR in nuclear and cytoplasmic fractions and normalized to levels of external RNA. **(C** and **D)** Luciferase activity in A549/DDP cells co-transfected with the wipe-type or mutant SNHG12 reporters (SNHG12-Wt or SNHG12-Mut) and (miR-181a mimics or miR-Con) or (anti-miR-181a or anti-miR-Con). **(E)** Cellular lysates from A549/DDP cells were used for RNA immunoprecipitation (RIP) with Ago2 antibody. Detection of SNHG12 and miR-181a using qRT-PCR. **(F)** The levels of SNHG12 were detected in A549/DDP cells after pcDNA-SNHG12 or pcDNA-SNHG12-Mut or SNHG12 siRNAs transfection. **(G)** qRT-PCR analysis was performed to detect the expression of miR-181a in A549/DDP cells after pcDNA-SNHG12 or pcDNA-SNHG12-Mut or SNHG12 siRNAs transfection. **(H)** Negative correlation between SNHG12 and miR-181a expressions in NSCLC tumor tissues. ^*^P < 0.05 versus control groups.

Recently, increasing evidence suggests that lncRNAs could act as miRNA sponges to negatively control miRNAs expression. Moreover, our results (Figure 1) showed that SNHG12 and miR-181a displayed opposing expression in NSCLC tissues and cell lines. Therefore, we speculated that SNHG12 may inhibit miR-181a by sponging miR-181a. To confirm our assumption, the online software starBase v2.0 was first used to predict candidate miRNAs for SNHG12 and possible recognition sequence of miRNAs on SNHG12. As expected, SNHG12 contains one conserved target binding site of miR-181a (Figure 2A). It was well known that the fuctional action of lncRNAs depended on its nuclear and cytoplasmic localization. As expected, our study found that SNHG12 was mainly located in cytoplasm (Figure 2B). To further validate the binding action between SNHG12 and miR-181a, luciferase reporter assay was performed in A549/DDP cells co-transfected with the wipe-type or mut SNHG12 reporters (SNHG12-Wt or SNHG12-Mut) and (miR-181a mimics or miR-Con) or (anti-miR-181a or anti-miR-Con). The results confirmed that miR-181a overexpression led to a dramatical reduction of the wild-type reporter activity in A549/DDP cells (Figure 2C), oppositely, a significant increase in the wild-type reporter activity when endogenous miR-181a was inhibited by anti-miR-181a (Figure 2D). However, the mutant reporter activities in all transfected cells had no significant changes (Figure 2C and 2D), indicating that the regulatory effect was dependent on the binding of SNHG12 and miR-181a. It is well known that SNHG12 may regulate miRNA expression through forming RNA-induced silencing complex (RISC). To further explore whether both SNHG12 and miR-181a were in the RISC complex, RIP experiments were performed on A549/DDP cell extracts using antibodies against Ago2. As shown in Figure 2E, SNHG12 and miR-181a were enriched in Ago2 pellets relative to control IgG. Then, the effect of SNHG12 on miR-181a expression was detected in A549/DDP cells transfected with (pcDNA-SNHG12 or pcDNA-SNHG12-Mut) or (si-SNHG12-1 or si-SNHG12-2). SNHG12 expression was significantly increased in pcDNA-SNHG12 or pcDNA-SNHG12-Mut transfecting cells compared with Vector transfection, while the SNHG12 level was remarkedly decreased in si-SNHG12-1 or si-SNHG12-2 transfecting cells when compared with si-Con transfection (Figure 2F). Moreover, miR-181a was downregulated by ectopic SNHG12, and its upregulation was observed after SNHG12 knockdown (Figure 2G). However, overexpression of SNHG12 with miR181a-binding site mutants had no significant effect on miR-181a expression (Figure 2G), which suggested that the inhibitory effect of SNHG12 on miR-181a depended on the miR181a-binding site. Furthermore, it was displayed that a negative correlation existed between SNHG12 and miR-181a in NSCLC tumor tissues (Figure 2H). Collectively, these data confirmed that SNHG12 directly suppressed miR-181a expression by sponging miR-181a in NSCLC.

### SNHG12 knockdown reverses MDR via improving drug-induced apoptosis in A549/DDP, A549/PTX and PC9/AB2 cells

**Figure 3:**
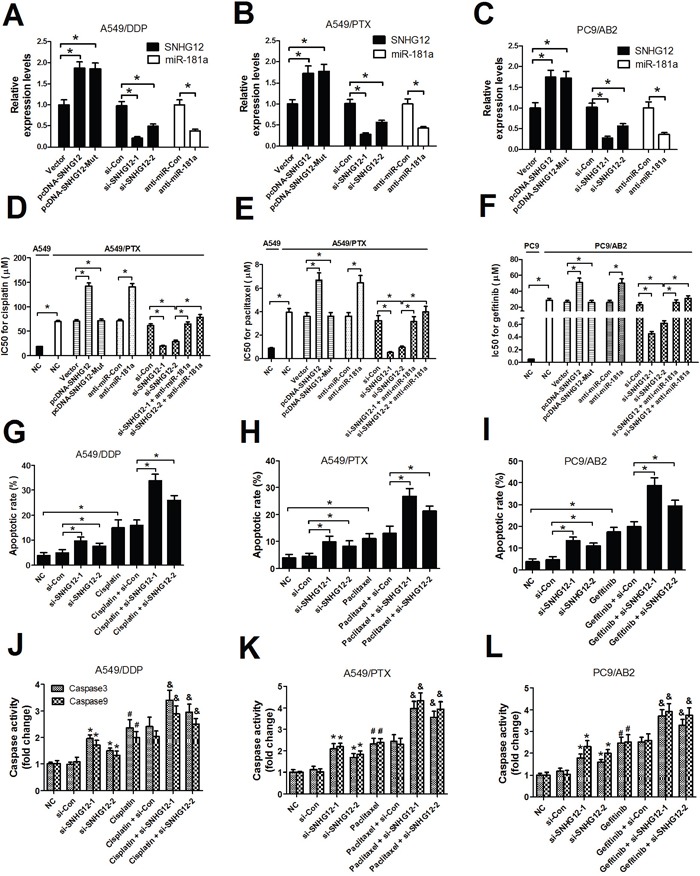
SNHG12 knockdown enhances sensitivity to DDP, PTX and AB2 in A549/DDP, A549/PTX and PC9/AB2 cells **(A-C)** The SNHG12 levels were determined by qRT-PCR analysis. **(D-F)** The IC50 values of DDP, PTX and AB2 in A549/DDP, A549/PTX and PC9/AB2 cells or their parent cells A549 and PC9 were determined by MTT assay. **(G-I)** Flow cytometry analysis was performed to examine the apoptotic rate in A549/DDP, A549/PTX and PC9/AB2 cells treated with cisplatin, paclitaxel or gefitinib for 48 h after transfection with SNHG12 siRNAs or si-Con. **(J-L)** Caspase activities were detected by a colorimetric assay kit in A549/DDP, A549/PTX and PC9/AB2 cells treated with cisplatin, paclitaxel and or gefitinib for 48 h after transfection with SNHG12 siRNAs or si-Con. The indicated concentration for cisplatin, paclitaxelor and gefitinib was 70 μM, 3.5 μM, and 25μM, respectively. ^*^P < 0.05 versus si-Con; ^#^*P* < 0.05 versus NC;^&^*P* < 0.05 versus (each drug + si-Con).

To determine the role of SHNG12 in MDR of NSCLC cells, pcDNA overexpression vectors-mediated gain-of-fuction or siRNA-mediated loss-of-function assays in A549 resistant strains (A549/PTX and A549/DDP) and PC9 resistant strain PC9/AB2 were performed. A549/PTX, A549/DDP and PC9/AB2 cells were transfected with (pcDNA-SNHG12 or pcDNA-SNHG12-Mut) or (si-SHNG12-1 or si-SHNG12-2) or anti-miR-181a or (si-SHNG12-1 or si-SHNG12-2 + anti-miR-181a). Firstly, the transfection efficiencies of SNHG12 overexpression vectors, SNHG12 siRNAs and anti-miR-181a were detected in A549/PTX, A549/DDP and PC9/AB2 cells. As shown in Figure 3A-3C, SNHG12 overexpression, SNHG12 knockdown and miR-181a inhibition were successful in A549/PTX, A549/DDP and PC9/AB2 cells. MTT assay was conducted to measure the IC50 value of A549 and PC9 cells, as well as their resistant cell strains (A549/PTX, A549/DDP and PC9/AB2). Results showed that the resistant strains A549/DDP (Figure 3A), A549/PTX (Figure 3B) and PC9/AB2 (Figure 3C) displayed higher IC50 of cisplatin (70 μM), paclitaxel (3.5 μM) and gefitinib (25 μM) than that of their respective parental cells (20μM/0.5μM/0.05μM), indicating the efficient resistance of the resistant cell strains to the anti-cancer drugs. Moreover, SNHG12 knockdown reversed the MDR of NSCLC cells, evidenced by a significant decrease in the IC50 of cisplatin, paclitaxel and gefitinib in si-SNHG12-transfected resistant cell strains compared with that of si-Con-transfected cells (Figure 3D-3F). Moreover, SNHG12 overexpression or miR-181a inhibition significantly increased the the MDR of NSCLC cells, while the mutant SNHG12 overexpression had no obvious effect on the MDR (Figure 3D-3F). However, miR-181a inhibition abolished si-SNHG12-mediated sensitivity of resistant cell strains to cisplatin, paclitaxel and gefitinib (Figure 3D-3F). All these results suggested that silence of SNHG12 reversed MDR of resistant cell strains in NSCLC by regulating miR-181a expression.

The above observations prompted us to investigate the possible mechanisms of SNHG12 in NSCLC MDR. Among the mechanisms responsible for MDR in various cancer cells, the repression of drug-induced apoptosis is particularly important. Therefore, flow cytometry analysis was performed to examine apoptosis in A549/DDP, A549/PTX and PC9/AB2 cells treated with cisplatin, paclitaxel and gefitinib after transfection with si-SNHG12-1 or si-SNHG12-2 or si-Con. The apoptotic rate of A549/DDP, A549/PTX and PC9/AB2 cells was indeed elevated by treatment with either anti-cancer drugs (cisplatin, paclitaxel and gefitinib) or (si-SNHG12-1 or si-SNHG12-2), while simultaneous SNHG12 downregulation and drug treatment led to significantly higher apoptotic cells (Figure 3G-3I). It is well documented that the abnormal expression of apoptosis-associated molecules, including caspases, is related to resistance to drug-induced apoptosis [17]. Therefore, the caspase3 and caspase9 activities were further investigated. As expected, silence of SNHG12 or anti-cancer drugs (cisplatin, paclitaxel or gefitinib) treatment promoted caspase3 and caspase9 activities in A549/DDP, A549/PTX and PC9/AB2 cells, however, combination of si-SNHG12 and drug treatment resulted in a significant increase in caspase3 and caspase9 activities in A549/DDP, A549/PTX and PC9/AB2 cells than any single treatment group (Figure 3J-3L). Considering the higher suppressive effect of si-SNHG12-1 on the MDR of NSCLC cells, si-SNHG12-1 was used in further experiments. Collectively, SNHG12 knockdown enhanced sensitivity to cisplatin, paclitaxel and gefitinib via improving drug-induced apoptosis in A549/DDP, A549/PTX and PC9/AB2 cells.

### miR-181a targets MAPK1 and MAP2K1, regulating p-MAPK1, p-MAP2K1 and slug expression

**Figure 4:**
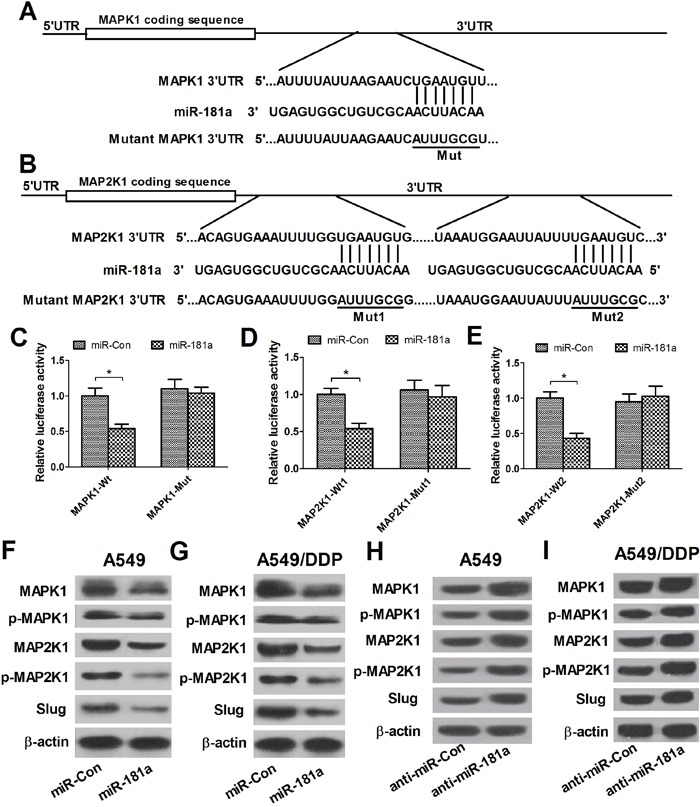
miR-181a targets MAPK1 and MAP2K1, regulating p-MAPK1, p-MAP2K1 and Slug expression **(A** and **B)** The putative miR-181a-binding sites within the 3’ UTR of MAPK1 and MAP2K1. **(C-E)** Luciferase activity in A549/DDP cells co-transfected with miR-181a or miR-Con and (MAPK1-Wt or MAPK1-Mut) or (MAP2K1-Wt1 or MAP2K1-Mut1) or (MAP2K1-Wt2 or MAP2K1-Mut2). **(F-I)** Western blot analysis was performed to detect the protein levels of MAPK1, p-MAPK1, MAP2K1, p-MAP2K1 and Slug in A594 and A549/DDP cells transfected with miR-181a or anti-miR-181a. ^*^P < 0.05 versus miR-con.

Increasing evidence suggests that miRNAs exert their functional role by regulating their target genes, which act as oncogenes or tumor suppressors involved in tumorigenesis. Therefore, the web-based miRNA databases TargetScan and miRBase were used to predict the possible targets of miR-181a. Among the targets, MAPK1 and MAP2K1, two crucial proteins in MAPK signaling pathways, were further studied based on the fact that MAPK pathways were confirmed to be involved in MDR in lung cancer [18]. As shown in Figure 4A and 4B, a target site for miR-181a were identified in the 3’ UTR of MAPK1, and two potential binding sequences for miR-181a was presented in the 3’ UTR of MAP2K1. Next, dual luciferase reporter assays were performed to confirm these targets in A549/DDP cells. As expected, the luciferase activities of the wild-type MAPK1 and MAP2K1 reporter vectors (MAPK1-Wt, MAP2K1-Wt1 and MAP2K1-Wt2) in A549/DDP cells were dramatically decreased by miR-181a mimics compared with that in miR-Con groups (Figure 4C-4E), while the luciferase activities of mutant reporter vectors were not significantly affected in all transfected cells. To further test the regulatory effect of miR-181a on MAPK/Slug pathway, western blot analysis was performed to examine the protein levels of MAPK1, p-MAPK1, MAP2K1, p-MAP2K1 and Slug. As shown in Figure 4F-4I, miR-181a overexpression significantly decreased the levels of MAPK1, p-MAPK1, MAP2K1, p-MAP2K1 and Slug while miR-181a inhibition elevated the levels of these proteins, indicating the inhibitory effect of miR-181a on MAPK/Slug pathway. Altogether, these results demonstrated that miR-181a suppressed p-MAPK1, p-MAP2K1 and Slug expression, crucial proteins of MAPK/Slug pathway, by targeting MAPK1 and MAP2K1.

### SNHG12 regulates MAPK/Slug pathway by sponging miR-181a

**Figure 5:**
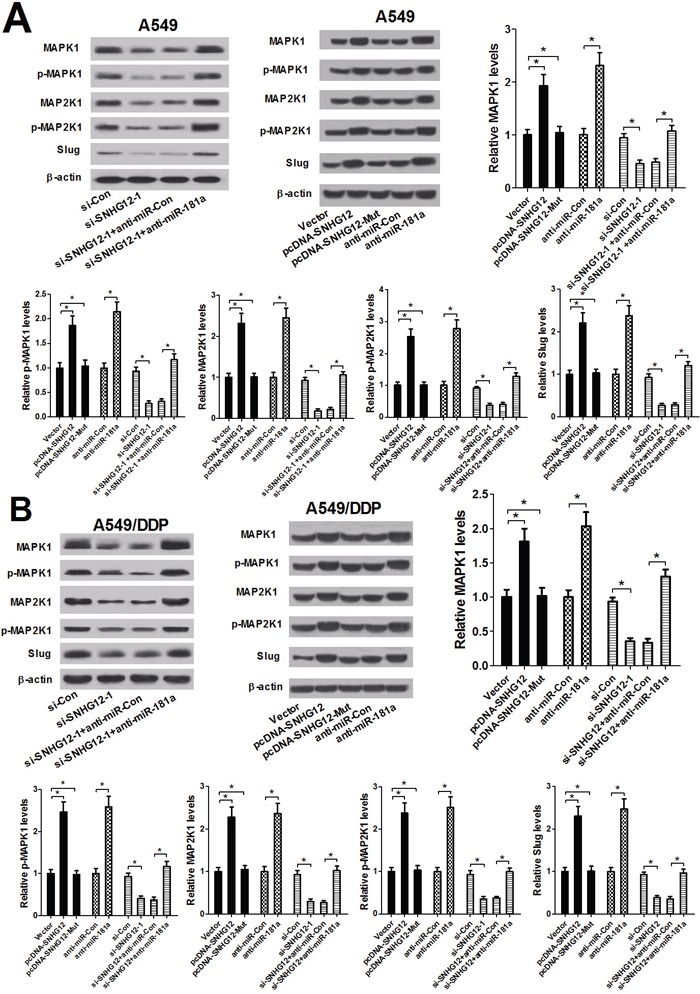
SNHG12 promotes MAPK/Slug pathway by sponging miR-181a A549 and A549/DPP cells were transfected with (si-Con or si-SNHG12) or (Vector or pcDNA-SNHG12 or pcDNA-SNHG12-Mut) or (anti-miR-181a or anti-miR-Con) or co-transfected with si-SNHG12-1 and anti-miR-181a or anti-miR-Con. **(A** and **B)** Western blot analysis was conducted to detect the levels of MAPK1, p-MAPK1, MAP2K1, p-MAP2K1 and Slug in A549 and A549/DPP cells. ^*^P < 0.05 versus control groups.

To further investigate whether SNHG12-miR-181a-MAPK/Slug regulatory axis existed in NSCLC cells, the levels of MAPK1, p-MAPK1, MAP2K1, p-MAP2K1 and Slug were determined by western blot analysis in A549 and A549/DDP cells that were transfected with (pcDNA-SNHG12 or pcDNA-SNHG12-Mut) or anti-miR-181a or si-SNHG12-1 or co-transfected with si-SNHG12-1 and miR-181a mimics. As shown in Figure 5A and 5B, SNHG12 knockdown led to a prominent reduction in the levels of MAPK/Slug-related proteins in A549 and A549/DDP cells. Moreover, SNHG12 overexpression or miR-181a inhibition significantly increased the protein levels of MAPK/Slug pathway, while the mutant SNHG12 overexpression had no obvious effect on these protein expression levels (Figure 5A and 5B). However, miR-181a inhibition by anti-miR-181a abolished the inhibitory effect of SNHG12 downregulation on MAPK/Slug pathway. These data suggested that silence of SNHG12 suppressed MAPK/Slug pathway by sponging miR-181a.

### Blockage of MAPK/Slug pathway reversed anti-miR-181a -mediated change in resistance to cisplatin and apoptosis in A549 cells

**Figure 6:**
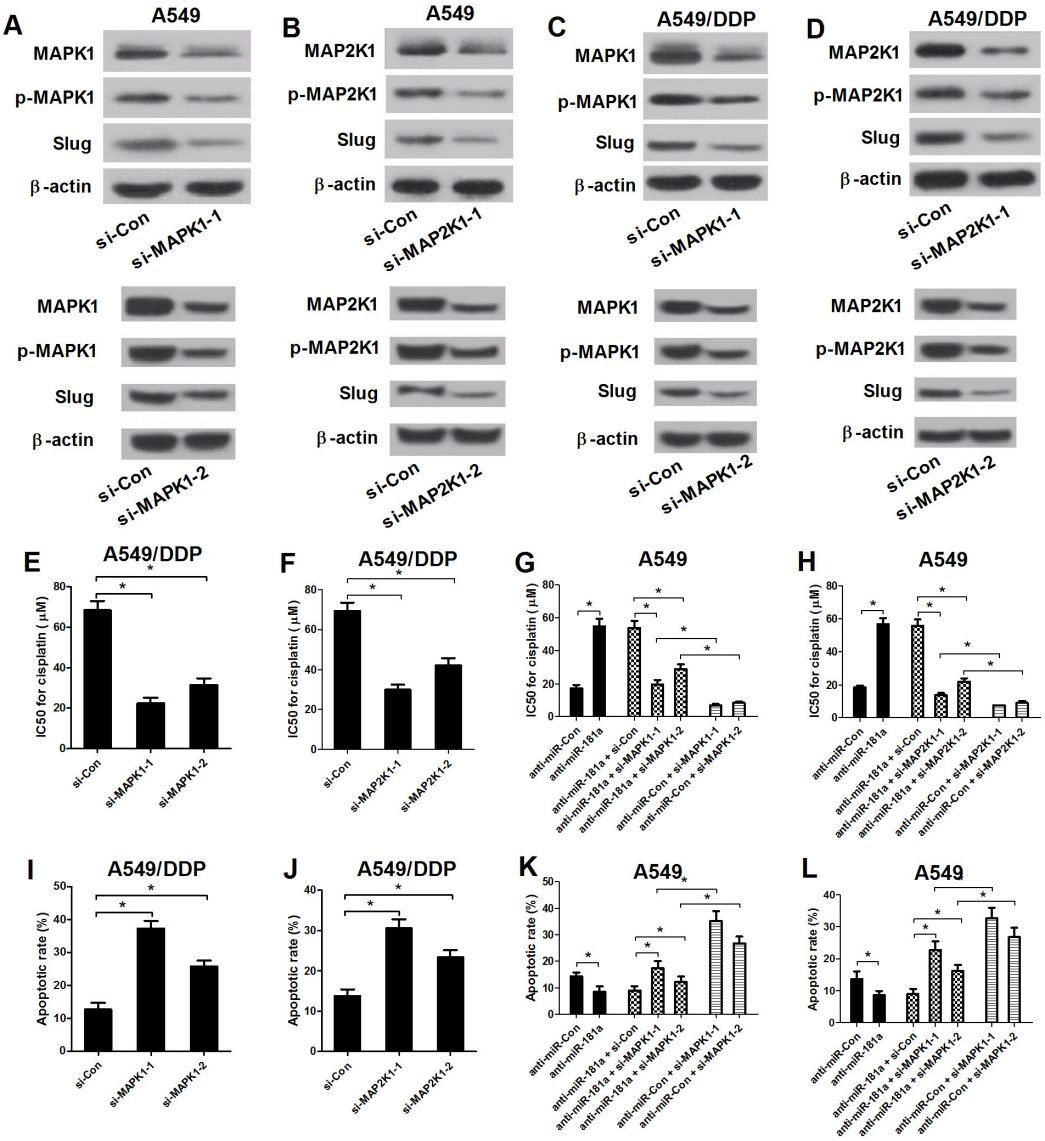
Inactivation of MAPK/Slug pathway reversed anti-miR-181a-mediated change in resistance to cisplatin and apoptosis in A549 cells **(A-D)** The levels of MAPK1, p-MAPK1, MAP2K1, p-MAP2K1 and Slug were examined by western blot analysis in A549 and A549/DPP cells transfected with si-Con, (si-MAPK1-1 or si-MAPK1-2) or (si-MAP2K1-1 or si-MAP2K1-2). **(E** and **F)** The IC50 values of DPP in A549/DPP cells transfected with si-Con, (si-MAPK1-1 or si-MAPK1-2) or (si-MAP2K1-1 or si-MAP2K1-2). **(G** and **H)** The IC50 values of DPP in A549 cells transfected with anti-miR-181a or in combination with (si-MAPK1-1 or si-MAPK1-2) or (si-MAP2K1-1 or si-MAP2K1-2). **(I** and **J)** Flow cytometry analysis was performed to examine the apoptotic rate in A549/DDP cells. **(K** and **L)** The apoptotic rate of A549 cells transfected with anti-miR-Con or in combination with (si-MAPK1-1 or si-MAPK1-2) or (si-MAP2K1-1 or si-MAP2K1-2). ^*^P < 0.05 versus control groups.

Emerging documents demonstrates that MAPK pathways are closely associated with MDR in cancers. To further confirm that the functional role of MAPK/Slug pathway in NSCLC drug resistance, A549 and A549/DPP cells were transfected with MAPK1 siRNAs or MAP2K1 siRNAs. Western blot analysis displayed an obvious decrease of MAPK1, p-MAPK1 and Slug expression by si-MAPK1-1 or si-MAPK1-2 transfection (Figure 6A and 6C), as well as an apparent decline of MAP2K1, p-MAP2K1 and Slug expression after trsnafection with si-MAP2K1-1 or si-MAP2K1-2 (Figure 6B and 6D) in A549 and A549/DPP cells, indicating that MAPK1 and MAP2K1 knockdown could effectively suppress MAPK/Slug pathway. Then, MTT assay and flow cytometry analysis were conducted to determine the IC50 values of cisplatin and cell apoptosis in (si-MAPK1-1 or si-MAPK1-2) or (si-MAP2K1-1 or si-MAP2K1-2) tranfected A549/DPP cells. Inhibition of MAPK/Slug pathway by (si-MAPK1-1 or si-MAPK1-2) (Figure 6E and 6I) or (si-MAP2K1-1 or si-MAP2K1-2) (Figure 6F and 6J) significantly enhanced cisplatin sensitivity, evidenced by decreased IC50 values of cisplatin and enhanced apoptosis in A549/DPP cells compared with si-Con groups. To further investigate whether the effect of MAPK/Slug pathway suppression on cisplatin resistance and apoptosis was regulated by miR-181a, A549 cells were transfected with anti-miR-181a or (anti-miR-181a or anti-miR-Con) in combination with si-MAPK1 or si-MAP2K1. MTT assay and flow cytometry analysis suggested that downregulation of miR-181a dramatically increased IC50 values of cisplatin (Figure 6G and 6H) and remarkedly suppressed cell apoptosis (Figure 6K and 6L) in A549 cells, which was abated by MAPK1 or MAP2K1 siRNAs- mediated inactivation of MAPK/Slug pathway. Collectively, miR-181a enhanced the cisplatin sensitivity of A549 cells through suppressing MAPK/Slug pathway by targeting MAPK1 and MAP2K1.

### SNHG12 knockdown enhances cisplatin sensitivity in NSCLC *in vivo*

**Figure 7:**
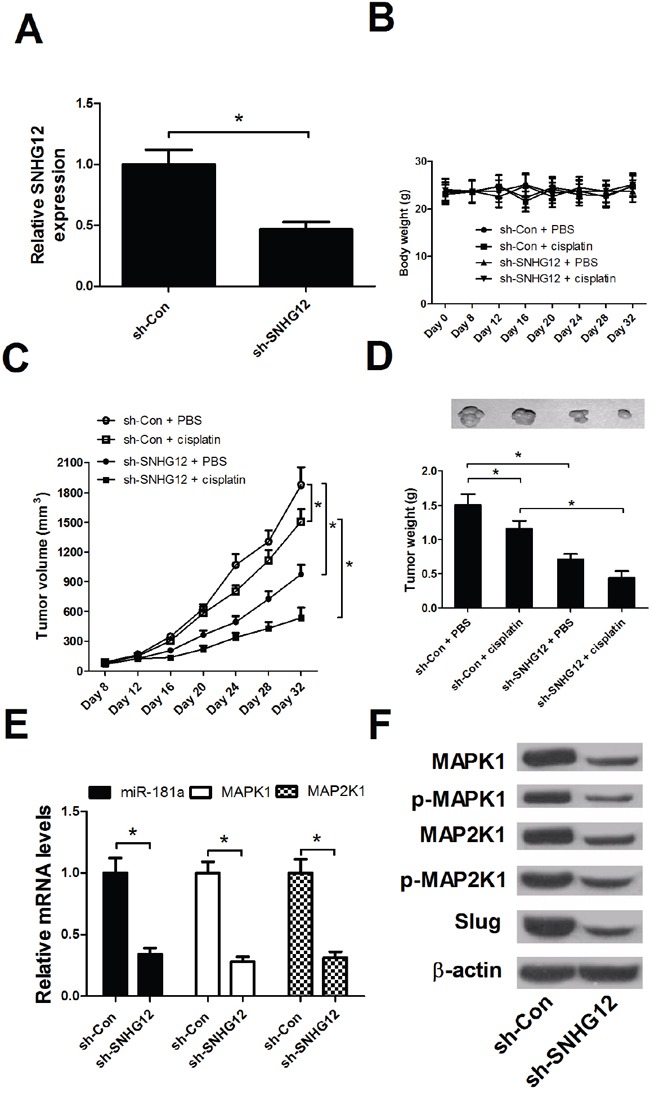
SNHG12 knockdown enhanced the DPP sensitivity of NSCLC *in vivo* A549/DDP-lenti-sh-SNHG12 or A549/DDP-sh-Con cells were injected subcutaneously into nude mice. At 8 days after inoculation, intraperitoneal injection of cisplatin (3.5 mg/kg, twice a week) was performed. **(A)** The knockdown efficienty of sh-SNHG12 was examined by qRT-PCR analysis. **(B)** The body weights of mice were detected every 4 days from 0 to 32 days. **(C)** The tumor volume was detected every 4 days from 8 to 32 days. **(D)** The tumor weight was measured and tumor appearance was photographed at 32 days after inoculation. **(E)** qRT-PCR analysis detected the miR-181a expression and MAPK1 and MAP2K1 mRNA levels in mice tumor tissues. **(F)** Western blot analysis was conducted to detect the levels of MAPK1, p-MAPK1, MAP2K1, p-MAP2K1 and Slug in mice tumor tissues. ^*^P < 0.05 versus control groups.

To further determine the effect of SNHG12 on the resistance of NSCLC cells to cisplatin *in vivo*, A549/DDP-lenti-sh-SNHG12 or A549/DDP-sh-Con cells were injected subcutaneously into mice to establish xenograft mouse model. At first, the knockdown efficiency of sh-SNHG12 was detected by qRT-PCR analysis. As shown in Figure 7A, SNHG12 knockdown was successful. Intraperitoneal injection of cisplatin was started at 8 days after transplantation when tumor volume reached about 100 mm^3^. The mice were killed and tumors were weighed after 32 days. The body weight of mice among all treated groups had no significant difference (Figure 7B). SNHG12 knockdown or cisplatin treatment led to a significant decrease in tumor volume and weight when compared with the control groups, and combination of SNHG12 knockdown and cisplatin treatment resulted in lower tumor volume and weight (Figure 7C and 7D). Then, the mechanism was further verified by qRT-PCR and Western blot. As shown in Figure 7E and 7F, SNHG12 knockdown dramatically increased miR-181a expression and significantly reduced MAPK1, p-MAPK1, MAP2K1, p-MAP2K1 and Slug protein levels in mice tissues. All these data demonstrated that SNHG12 knockdown enhanced sensitivity of NSCLC cells to cisplatin *in vivo*.

## DISCUSSION

The developments of platinum-based chemotherapy and targeted therapies for EGFR-sensitive and ALK-positive patients have been regarded as milestones for lung cancer treatment [19]. However, acquired resistance is a major barrier occurring in lung cancer chemotherapy. Emerging evidence suggests that lncRNAs may play an important role in the development of chemoresistance in lung cancers. For instance, HOTAIR was upregulated in small cell lung cancer multidrug-resistant cell lines (H69AR and H446AR), and depletion of HOTAIR enhanced H69AR and H446AR cell sensitivity to anticancer drugs through increasing cell apoptosis and cell cycle arrest by inhibiting DNMT1 and DNMT3b expression and reducing HOXA1 methylation [20]. Wu *et al.* demonstrated that linc00635-001 silencing accompanied by gefitinib treatment sensitized lung cancer cells HCC827-8-1 to gefitinib-induced cytotoxicity through the suppression of Akt activation [21]. Conversely, MEG3 was low-expressed in A549/DDP cells, and overexpression of MEG3 partially reversed the resistance of A549/DDP cells to cisplatin through the regulation of p53 and Bcl-xl expression [22]. Another study revealed that downregulation of lincAK126698 depressed cisplatin-induced apoptosis in A549 cells through altered Wnt signaling by inhibiting naked cuticle homolog 2 expression and promoting β-catenin expression [23]. All these publications demonstrated that lncRNAs could contribute to the sensitivity or resistance of lung cancer cells to anti-cancer drugs, indicating the crucial roles of lncRNAs in the development of lung cancer chemoresistance.

Recently, a novel lncRNA SNHG12 have been reported to be upregulated and play an oncogenic role in many kinds of cancers. SNHG12 was first identified to be significantly upregulated in endometrial cancer, and inhibition of SHNG12 expression led to proliferation suppression and apoptosis induction in endometrial cancer cells [24]. Ruan *et al.* revealed that SNHG12 contributed to cell proliferation and migration in human osteosarcoma cells by upregulating angiomotin expression [25]. Another study demonstrated that c-MYC-induced SNHG12 upregulation promoted cell proliferation and suppressed apoptosis in triple-negative breast cancer [26]. SNHG12 was also clarified to promote cell migration by regulating MMP13 expression in breast cancer [26]. In hepatocellular carcinoma, SNHG12 promoted tumorigenesis and metastasis by acting as an endogenous miR-199a/b-5p sponge to regulate the expression of MLK3 and affect the NF-κB pathway [27]. In the present study, we found that SNHG12 was upregulated in NSCLC tissues and cell lines. The level of SNHG12 was elevated in NSCLC resistant cell strains (A549/DDP, A549/PTX and PC9/AB2) compared with their respective parental cell line. Moreover, knockdown of SNHG12 reversed the resistance of A549/DDP to cisplatin, A549/PTX to paclitaxel and PC9/AB2 to gefitinib. The repression of anti-cancer drug-induced apoptosis was one of the main mechanisms responsible for MDR in cancers. Consistent with this theory, our study found SNHG12 silencing enhanced cisplatin-induced A549/DDP apoptosis, paclitaxel-induced A549/PTX apoptosis and gefitinib-induced PC9/AB2 apoptosis. Moreover, miR-181a inhibition could prevent cisplatin-induced A549 apoptosis, which could be reversed by MAPK1 or MAP2K1 knockdown. All these data revealed that the SNHG12-miR-181a-MAPK/Slug axis enhanced the drug sensitivity of NSCLC cells by promoting drug-induced cell apoptosis. Furthermore, SNHG12 knockdown enhanced cisplatin sensitivity of NSCLC *in vivo*. To our knowledge, this study is the first to identify the promotive role of SNHG12 in MDR, facilitating the development of novel therapeutic strategies for resistant NSCLC.

Mechanically, mounting lncRNAs can act as ceRNAs to de-repress miRNAs target genes by sponging special miRNAs [28, 29]. An inverse correlation between SNHG12 and miR-181a expressions stimulated our interest to determine whether a ceRNA mechanism existed between SNHG12 and miR-181a. Bioinformatics analysis and luciferase reporter assays confirmed that the interaction between SNHG12 and miR-181a in A549/DDP cells. qRT-PCR analysis further confirmed that SNHG12 negatively modulated miR-181a expression in A549/DDP cells. Furthermore, SNHG12 was confirmed to release miR-181a target genes MAPK1 and MAP2K1, by sponging miR-181a in A549/DDP cells. All these findings illuminated that SNHG12 acted as a ceRNA to regulate MAPK1 and MAP2K1 by sponging miR-181a in NSCLC. Similarly, a previous study also revealed SNHG12 de-suppress mixed-lineage protein kinase 3 (MLK3) by sponging miR-199a/b-5p in hepatocellular carcinoma [27]. Functionally, SNHG12 knockdown induced sensitivity of A549/DDP to cisplatin, A549/PTX to paclitaxel and PC9/AB2 to gefitinib by sponging miR-181a. Moreover, miR-181a inhibition led to MDR of A549 cells by targeting MAPK1 and MAP2K1. Overall, downregulation of SNHG12 reversed MDR of NSCLC resistant cells through de-repressing MAPK1 and MAP2K1 by sponging miR- 181a.

MAPK1 and MAP2K1 were previously reported as the target genes of miR-181a in salivary adenoid cystic carcinoma [30], in agreement with our study. They are members of the mitogen-activated protein kinase (MAPK) group, which plays a crucial role in multiple signaling cascades, including MAPK/Slug pathway. Our study found that MAPK1 and MAP2K1 knockdown suppressed p-MAPK1, p-MAP2K1 and Slug involved in MAPK/Slug pathway. Moreover, MAPK/Slug pathway inhibition by si-MAPK1 and si-MAP2K1 enhanced the cisplatin sensitivity and apoptosis of A549/DPP cells. In accordance with to our finding, a previous study indicated that inactivation of MAPK/Slug signals enhanced the sensitivity of HL-60 leukemia cells to cytarabine by upregulating P53 up-regulated modulator of apoptosis (PUMA) [31]. Additionally, many studies have proved Slug significantly inhibits PUMA-induced apoptosis [32, 33]. Therefore, we speculate inhibition of MAPK/Slug pathway may induce apoptosis of A549/DPP cells by upregulating PUMA, which is need further research to elucidate.

In summary, our study found upregulated SNHG12 and downregulated miR-181a in NSCLC tumor tissues and cell lines. Functionally, knockdown of SNHG12 enhanced the sensitivity of NSCLC resistant cells *in vitro* and *in vivo*. Mechanically, SNHG12 acted as an oncogene in NSCLC MDR via inducing apoptosis through modulating MAPK/Slug pathway by sponging miR-181a and releasing MAPK1 and MAP2K1. Our findings revealed a SNHG12-miR-181a-MAPK/Slug axis in NSCLC MDR, providing a new modulation strategy to overcome chemoresistance of NSCLC.

## MATERIALS AND METHODS

### Tissue specimens and cell lines

Twenty-two NSCLC tissue samples and matched normal adjacent tissue samples were obtained from lung cancer patients undergoing surgery, with the informed consents of all patients. This study was approved by the Ethic Review Committees of Huaihe Hospital of Henan University. No patients experienced radiotherapy or chemotherapy prior to surgery. Pathological assessments of tissues were performed by pathologists at the Department of Pathology, Huaihe Hospital of Henan University. The excised tissues were stored at -80°C for further analysis.

Immortalized human bronchial epithelial cell line HBE [34], NSCLC cell line PC9, NSCLC cell line A549 and DDP-resistant cell line A549/DDP were purchased from Cell bank of Chinese Academy of Sciences (Shanghai, China). NSCLC cell lines H1299 and H358 were purchased from the American Type Culture Collection (ATCC). Paclitaxel-resistant cell line A549/PTX and gefitinib-resistant cell line PC9/AB2 were established as previously described [35, 36]. All cells were cultured in RPMI-1640 medium (Invitrogen, Carlsbad, CA, USA) containing 10% fetal bovine serum (Invitrogen) and 1% penicillin/streptomycin at 37°C under humidified air with 5% CO_2_. In order to maintain drug-resistant phenotype, the medium of A549/DDP, A549/PTX, or PC9/AB2 was additionally supplemented with 2 mg/ml DDP (Sigma-Aldrich, St. Louis, USA), 100 ng/ml PTX (Sigma-Aldrich), or 2 μmol/l gefitinib (Sigma-Aldrich).

### Quantitative real-time PCR (qRT-PCR)

Total RNA was extracted from NSCLC tissue samples or cell lines using TRIzol reagent (Invitrogen). Reverse transcription was conducted using the First Strand cDNA synthesis kit (Takara, Otsu, Shiga, Japan). qRT-PCR was carried out on the iCycler IQ multi-color Detection System (Bio-Rad, Hercules, California, USA) using an IQ SYBR-Green Supermix (Bio-Rad). The primer sequences used for amplification are as follow: SNHG12, 5’-TCT GGT GAT CGA GGA CTT CC-3’ (forward) and 5’-ACC TCC TCA GTA TCA CAC ACT-3’ (reverse); GAPDH, 5’-CCC ACT TGA AGG GTG GAG CCA A-3’ (forward) and 5’-TGG CAT GGA CTG TGG TCA TGA-3’ (reverse); miR-181a, 5’-AAC ATT CAA CGC TGT CG-3’ (forward) and 5’-AAC TGT GTC GTG GAG-3’ (reverse); U6, 5′-GCT TCG GCA GCA CAT ATA CTA A-3’ (forward) and 5′-AAC GCT TCA CGA ATT TGC GT-3’ (reverse). Fold changes of SNHG12 and miR-181a were analyzed using the 2^-ΔΔCt^ method by normalizing to GAPDH and U6 snRNA, respectively.

### Transfection

miR-181a mimics, scramble miRNA control (miR-Con), miR-181a inhibitors (anti-miR-181a) and its nonspecific control (anti-miR-Con) were purchased from GenePharma (Shanghai, China). The siRNAs against SNHG12 (si-SNHG12-1 and si-SNHG12-2), siRNAs against MAPK1 (si-MAPK1-1 and si-MAPK1-2), siRNAs against MAP2K1 (si-MAP2K1-1 and si-MAP2K1-2) and non-targeting control sequence (si-Con) were synthesized by RiboBio (Guangzhou, China). The siRNA oligonucleotide sequences targeting SNHG12 were as follows: si-SNHG12-1, sense 5’-GCAGUGUGCUACUGAACUUTT-3’, and antisense 5’-AAGUUCAGUAGCACACUGCTT-3’; si-SNHG12-2, sense 5’-UGUGAUACUGAGGAGGUGATT-3’, and antisense 5’-UCACCUCCUCAGUAUCACATT-3’. For SNHG12 overexpression, the wild or miR-181a biding site mutant SNHG12 sequence was amplified and inserted into pcDNA 3.1 vector from Ribobio (Guangzhou, China). Cells transfection with oligonucleotide or plasmids was performed using Lipofectamine 2000 (Invitrogen) at appropriate concentrations.

### Drug sensitivity assay

The drug sensitivity assay was performed as described previously [37]. Briefly, 5 × 10^3^ transfected cells were seeded into 96-well plate. After incubation for 24 h, the anti-cancer drug (cisplatin, paclitaxel or gefitinib) or dimethyl sulfoxide (DMSO) was added into each well with the appropriate concentration gradient for each drug (for cisplatin, from 1.25 to 320 μM; for paclitaxel, from 0.25 to 16 μM; for gefitinib, from 0.1 to 102.4 μM). After 48 h, MTT assay (Sigma, St Louis, MO, USA) was performed to detect cell viability by measuring absorbance at 490 nm on a spectrophotometer. The IC50 values (the drug concentration producing 50% inhibition of growth) for different drugs were then calculated using GraphPad Prism.

### Apoptosis assay

Transfected cells were seeded in 96-well plates at a 5×103/well. After 24 h, cells were further treated with cisplatin (70 μM), paclitaxel (3.5 μM), gefitinib (25 μM) or DMSO for 48 h. Then, cells were harvested and stained with FITC-Annexin V and Propidium iodide (PI) using an Annexin-V-FITC apoptosis detection kit (BD Bioscience, Franklin Lakes, NJ, USA). Apoptosis data was acquired and analyzed by the FACScan flow cytometer (BD Bioscience) with CellQuest software (BD Bioscience).

### Caspase activity assay

The activities of caspase3 and caspase9 were measured by a colorimetric assay kit (R&D Systems Inc., Minneapolis, MN, USA). Briefly, transfected cells were treated with 70 μM cisplatin, 3.5 μM paclitaxel, 25 μM gefitinib or DMSO for 48 h. Then cells were collected, washed with PBS and lysed with iced lysis buffer provided by the manufacture. After centrifugation, clear lysates corresponding to 50 μg of protein were incubated with reaction buffer containing enzyme-specific colorigenic substrates for caspase3 and caspase9 at 37°C for 1 h. A microplate reader (BioTek Instruments, Winooski, VT, USA) was used to detect fold change in absorbance at 405 nm.

### Nuclear and cytoplasmic RNA extraction

RNA was extracted from the nucleus and cytoplasm according to the Invitrogen nuclear extraction protocol. Cells were resuspended in 500μl 1x Hypotonic Buffer. After 15 min incubation on ice, 10% NP40 detergent was added, and the samples were vortexed. Then, the homogenate was centrifuged for 10 minutes at 3,000 rpm at 4°C. The RNA from the pellet, containing the nuclear fraction, was extracted by the Tri Reagent method. The RNA from the supernatant, containing the cytoplasmic fraction, was extracted by the Phenol-Chloroform method.

### Luciferase reporter assay

The sequence fragment of SHNG12 (SHNG12-Wt), MAPK1 (MAPK1-Wt) 3’UTR and MAP2K1 3’UTR (MAP2K1-Wt1 or MAP2K1-Wt2) containing the putative target sites for miR-181a were amplified and inserted into the pGL3-reporter-vector (Promega, Madison, WI, USA). The mutant miR-181a target sites for SHNG12 (SHNG12-Mut), MAPK1 3’UTR (MAPK1-Mut) and MAP2K1 3’UTR (MAP2K1-Mut1 or MAP2K1-Mut2) were generated by using the QuikChange II Site-Directed Mutagenesis Kit (Stratagene, La Jolla, CA, USA). For confirming the binding interaction between SHNG12 and miR-181a, A549/DDP cells were co-transfected with (miR-181a mimics or miR-Con) or (anti-miR-181a or anti-miR-Con) and pGL3-SHNG12-Wt or pGL3-SHNG12-Mut. For verifying that MAPK1 and MAP2K1 were targets of miR-181a, (pGL3-MAPK1-Wt or pGL3-MAPK1-Mut) or (pGL3-MAP2K1-Wt1 or pGL3-MAP2K1-Mut1) or (pGL3-MAP2K1-Wt2 or pGL3-MAP2K1-Mut2) were transfected into A549/DDP cells in combination with miR-181a mimics or miR-Con. pRL-SV40 plasmid (Promega) carrying *Renilla* luciferase was also co-transfected to cells for standardizing transfection efficiency. The activities of *Renilla* luciferase and firefly luciferase were detected 48 h after transfection using Dual-Luciferase reporter assay system (Promega). The firefly luciferase activity was normalized to Renilla luciferase activity.

### RNA immunoprecipitation (RIP) assay

RNA immunoprecipitation was performed using the Magna RIP Kit (Millipore, Billerica, MA, USA). Briefly, A549/DDP cells were lysed in RIP lysis buffer, then the cell extract was incubated with RIP buffer containing magnetic beads conjugated with human anti-Ago2 antibody, positive control anti-snRNP70 or negative control normal mouse IgG (Millipore). To digest the protein, samples were incubated with Proteinase K with shaking. Then immunoprecipitated RNA was isolated. Finally, the levels of SNHG12 and miR-181a in the precipitates were detected by qRT-PCR.

### Western blot analysis

After transfections for 48 h, cells were collected and lysed using RIPA buffer (Beyotime, Shanghai, China). Equal amount of total protein from each sample was separated on SDS-PAGE and then transferred to nitrocellulose membranes (Millipore, Bedford, MA, USA). Then, the membranes were incubated with the primary antibodies including anti-MAPK1 (Upstate Biotechnology, Lake Placid, NY, USA), anti-p-MAPK1, anti-MAP2K1, anti-p-MAP2K1, anti-Slug (Cell Signaling Technology, Danvers, MA, USA), or anti-β-actin (Sigma). The protein bands were visualized using enhanced chemiluminescence (Amersham Pharmacia Corp, Piscataway, NJ, USA) and quantified by ImageJ software (National Institutes of Health, Bethesda, MD, USA).

### Xenograft in nude mice

Recombinant lentiviruses containing shRNA-control (sh-Con) or shRNA-SNHG12 (sh-SNHG12) (5’-TGCACTAGCTGGCATCACCGC-3’) were purchased from the GeneChem Company (Shanghai, China) and stably transfected into A549/DDP cells, termed as A549/DDP-lenti-sh-Con or A549/DDP-lenti-sh- SNHG12. For *in vivo* experiments, 5-week-old male athymic nude mice (BALB/c nu/nu) were maintained in specific-pathogen-free conditions with a light/dark cycle of 12/12 h and were given standard chow diet and water. 1×10^7^ A549/DDP-lenti-sh-Con or A549/DDP-lenti-sh-SNHG12 cells suspending in 100 μl PBS were injected subcutaneously to the right flank of mice. At 8 days post-inoculation, the mice (n=6 per group) received intraperitoneal injections of cisplatin (3.5 mg/kg, twice a week) or PBS (as a control) [38]. Tumor growth was measured with calipers every 4 days. Tumor volumes were calculated by the following equation: tumor volume (mm^3^) = 0.5 × length (mm) × width (mm) × width (mm). Mice were killed 32-day post transplantation for tumor weight analysis. All animal experiments were performed with the approval of the Institutional Animal Care and Use Committee of Huaihe Hospital of Henan University.

### Statistical analysis

All experiments were performed at least three times. Quantitative data were expressed as mean ± SD and analyzed using SPSS 19.0 statistics software (SPSS, Chicago, IL, USA). Significant differences among groups were determined by Student's *t*-test or one-way ANOVA. *P* < 0.05 was deemed to be statistically significant.

## Abbreviations

SNHG12: small nucleolar RNA host gene 12
NSCLC: non-small cell lung cancer
MDR: multidrug resistance
p-MAPK1: phosphorylated MAPK1
p-MAP2K1: phosphorylated MAP2K1
EGFR: epidermal growth factor receptor
miRNAs: microRNAs
lncRNAs: long non-coding RNAs
PI: propidium iodide
MAPK: mitogen-activated protein kinase
